# The Closing of the Year

**Published:** 1901-12

**Authors:** 


### THE CLOSING OF THE YEAR.

1901 will long be remembered by those engaged in the various branches of the live-stock industry. It has been the year that witnessed the end of the long period of depression in this field, which had covered more than five years before it reached its termination. It will be equally well remembered by the veterinary profession as the one year, above all others, that fully determined the sphere of the auto-wagons and auto-trucks, and brought in its train a return of the true values in horses. It gave to the veterinary profession a fresh stimulus and renewed hopes, aims, and purposes, and restored to our colleges a support that had for nearly two years grown less and less. These periods of severe depression are not so likely to recur in the future, as we learn the lessons they teach and rear up the safeguards that are to save us from the trials and distress that follow in their pathway. They point the way of their prevention, and teach valuable lessons to our ranks, our schools, and through us to all the branches of the wonderful animal industry of our land.

